# Editorial: Pain and Depression

**DOI:** 10.3389/fpsyg.2022.865071

**Published:** 2022-03-30

**Authors:** Qing Zhao, Yazhuo Kong, Li Wan

**Affiliations:** ^1^CAS Key Laboratory of Mental Health, Institute of Psychology, Beijing, China; ^2^Department of Psychology, University of Chinese Academy of Sciences, Beijing, China; ^3^Department of Pain Management, The State Key Clinical Specialty in Pain Medicine, The Second Affiliated Hospital of Guangzhou Medical University, Guangzhou, China

**Keywords:** pain, depression, pain–depression comorbidity, neuroinflammation, non-opioid interventions

This special issue, *Pain and Depression*, aims to collect and present the current scholars' knowledge on pain–depression comorbidity. Pain and depression are intertwined and undermine the wellbeing of millions around the world. Nevertheless, the reasons for the association of pain and depression are still unclear. We consider that: (1) Pain–depression comorbid conditions might correspond to physiological–psychological spectral manifestations of underlying health problems (Von Korff and Simon, 1996); (2) Pain and depression could be in a bilateral causal loop (Nicolson et al., 2009). That is, pain might induce depression (Brown, 1990), and depression could increase people's sensitivity to pain as well as the risk of developing chronic pain (Hermesdorf et al., 2016); (3) The causal loop of pain and depression might evolve into a vicious circle without effective medical and mental intervention (Rosemann et al., 2007). A better summarization of the relationship between pain and depression could be the first step toward decoding the nature of the pain–depression loop.

Therefore, we presented the special issue of *Pain and Depression* to the Frontiers in Psychology (Health Psychology, Emotion Science, and Neuropsychology) and the Frontiers in Behavioral Neuroscience (Pathological Conditions). From the 8^th^ July 2019 to the 20^th^ September 2021, with dedications by 65 authors and 21 reviewers, 11 manuscripts have been published. These studies included three qualitative and quantitative reviews (Campos et al.; Davis et al.; Du et al.), three self-report studies (Boring et al.; Catalá et al.; Peng et al.), two behavioral examinations (Li et al.; Guo et al.), a brain imaging investigation (Jami et al.), a cross-species experiment (Zhang et al.), and a theoretical proposal (Cukić).

Du et al. reviewed the 100 top-cited studies of the pain-depression relationship. They found that 47% of the studies supported a causal effect from pain to depression (i.e., pain → depression), while 9% suggested the opposite relationship (i.e., depression → pain; see Table 1 of Du et al.). Meanwhile, 23% of the studies presented a positive correlation between pain and depression, but 3% reported an insignificant correlation (Du et al.).

From the physical viewpoint, Campos et al. proposed that neuroinflammation underpinned the pain–depression comorbidity. Through a cross-species investigation, Zhang et al. emphasized the role of inflammatory molecules in causing radicular pain. According to Campos et al., the key to alleviating the pain–depression symptoms could be the pharmacological modulation of inflammatory mediators (e.g., cytokines). Alternatively, Cukić sustained the efficiency of repetitive transcranial magnetic stimulation (rTMS) and transcranial direct current stimulation (tDCS) in treating depression featured by aberrant fronto-limbic functional connectivity. Essentially, abnormal neurotransmission in the mesolimbic dopamine system (i.e., a network connecting the prefrontal cortex and the limbic system) could underpin comorbidities among pain, depression, and addiction (Serafini et al., 2020). Therefore, identifying effective, specific, and alternative non-opioid therapies for patients with pain and depression could be increasingly important (Serafini et al., 2020), specifically during the opioid epidemic (e.g., Rogers et al., 2021).

From the psychological viewpoint, Boring et al. indicated that the mediator between pain and depression could be the feeling of shame (e.g., self-doubt and self-criticism). With healthy volunteers, they found that previous experiences of “pain invalidation” from family, friends, and medical professionals might lead to the feeling of shame, which in turn could give rise to depression (Boring et al.). The above study highlighted the importance of empathy for pain in society (Jami et al.). Moreover, Li et al. observed that healthy volunteers with capsaicin-induced pain could show disturbed processing (e.g., a longer reaction time) of emotions, especially of sadness. It should be noted that the conscious and unconscious attentional biases for negative emotions are a risk marker of depression (Watters and Williams, 2011). These studies hinted at the necessity of psychological interventions for patients with pain and depression. For example, Davis et al. listed a spectrum of non-pharmacological interventions (e.g., mindfulness-based therapy and guided imagery with relaxation) for patients with inflammatory bowel disease, anxiety, and depression.

Intriguingly, Catalá et al. reported that although female patients living in rural areas suffered more pain symptoms, their psychological wellbeing (e.g., pain acceptance and mental fatigue) was better than their urban counterparts. More importantly, Guo et al. found that Chinese university students' depression was negatively correlated with their forced vital capacity. These studies suggested that depression symptoms might be related to insufficient oxygen intake (Miravitlles et al., 2014), and indicated the possibility of administering oxygen-enriched air while treating depressed patients (Bloch et al., 2021). Referencing Catalá et al., rural areas with advanced clinical facilities could be ideal for recovering from pain–depression comorbidity.

## For the Future

Pain (Clauw et al., 2020) and depression (Peng et al.) are of growing concern in the current and post-COVID-19 eras. This special issue presented a glimpse of the pain–depression relationship. It is essential to keep on investigating the etiology of pain–depression comorbidity (e.g., neurological, neurohumoral, psychological, and physical mechanisms), identifying moderators of the comorbidity (e.g., living conditions, socioeconomic status, and view of life), and exploring the effective non-opioid pharmacological, physiological, and psychological treatments for the issue (e.g., acupuncture and biofeedback; Nicolson et al., 2009; Lee et al., 2019). Notably, males tend to conceal more physical and mental suffering than females to protect their masculinity (Shi et al., 2021). Therefore, the pain–depression causal relationship, its moderators, and its corresponding intervention could vary according to sex. Finally, the importance of empathy in helping patients suffering from pain, depression, and addiction deserves attention in the Age of Empathy (Jami et al.; De Waal, 2009).

## Author Contributions

QZ wrote the first draft of the editorial. YK and LW conducted the editorial revision. All authors contributed to the article and approved the submitted version.

## Conflict of Interest

The authors declare that the research was conducted in the absence of any commercial or financial relationships that could be construed as a potential conflict of interest.

## Publisher's Note

All claims expressed in this article are solely those of the authors and do not necessarily represent those of their affiliated organizations, or those of the publisher, the editors and the reviewers. Any product that may be evaluated in this article, or claim that may be made by its manufacturer, is not guaranteed or endorsed by the publisher.
